# Disproportionality Analysis of Adverse Events Associated with IL-1 Inhibitors in the FDA Adverse Event Reporting System (FAERS)

**DOI:** 10.3390/ph18121827

**Published:** 2025-12-01

**Authors:** Jingjing Lei, Zhuoran Lou, Yuhua Jiang, Yue Cui, Sha Li, Jinhao Hu, Yeteng Jing, Jinsheng Yang

**Affiliations:** Institute of Basic Theory for Chinese Medicine, China Academy of Chinese Medical Sciences, Beijing 100700, China; leijingjing137@163.com (J.L.); zyxlouzhuoran@163.com (Z.L.); jiangyuhua1996@outlook.com (Y.J.); cuiyue19981225@163.com (Y.C.); sjzlisa@163.com (S.L.); hujinhao0210@163.com (J.H.); jytxq1015@163.com (Y.J.)

**Keywords:** IL-1 inhibitors, adverse events, FAERS, disproportionality analysis

## Abstract

**Background**: Interleukin-1 (IL-1) inhibitors are approved for the treatment of various inflammatory diseases associated with immune system abnormalities. However, large-scale real-world studies to assess their security are still limited. Therefore, a pharmacovigilance study was conducted based on the data from the U.S. Food and Drug Administration (FDA) Adverse Event Reporting System (FAERS). **Methods**: Adverse events (AEs) linked to IL-1 inhibitors were analyzed using the FAERS database from Q1 2004 to Q3 2024. Risk signals were identified through disproportionality analysis algorithms, including reporting odds ratio (ROR), proportional reporting ratio (PRR), Bayesian confidence propagation neural network (BCPNN), and multi-item gamma Poisson shrinker (MGPS). **Results**: Among 17,670 AE reports where an IL-1 inhibitor was the “primary suspected” drug, 27 significant system organ classes (SOCs) were identified. Notable signals included infections and infestations (ROR: 2.31, 95% CI: 2.25–2.37) and congenital, familial, and genetic disorders (ROR: 2.26, 95% CI: 2.05–2.48). At the preferred term (PT) level, 263 significant AE signals were detected, such as pyrexia (ROR: 5.27, 95% CI: 5.03–5.53), nasopharyngitis (ROR: 2.31, 95% CI: 2.10–2.54), and injection site erythema (ROR: 6.09, 95% CI: 5.67–6.55). Importantly, we also identified less common or previously unreported AEs, including cardiac disorders (e.g., postural orthostatic tachycardia syndrome with anakinra; pulmonary valve incompetence with rilonacept) and endocrine disorders (e.g., secondary adrenocortical insufficiency with canakinumab). Furthermore, 36.33% of cases emerged after more than 360 days of treatment with IL-1 inhibitors. **Conclusions**: This study revealed real-world safety data on IL-1 inhibitors, providing important insights to enhance the clinical use of IL-1 inhibitors and minimize potential AEs.

## 1. Introduction

Interleukin-1 (IL-1), a key cytokine in innate immunity and inflammation, includes two main isoforms, IL-1α and IL-1β, which activate the IL-1 receptor type 1 (IL-1R1) to initiate NF-κB and MAPK signaling pathways [1]. Excessive IL-1 production leads to pathological inflammation in autoimmune and autoinflammatory diseases such as rheumatoid arthritis (RA), systemic juvenile idiopathic arthritis (sJIA), and cryopyrin-associated periodic syndromes (CAPS) [2,3,4]. Three FDA-approved IL-1 inhibitors—anakinra (a recombinant IL-1 receptor antagonist), canakinumab (an anti-IL-1β monoclonal antibody), and rilonacept (an IL-1 trap fusion protein)—are utilized to mitigate IL-1-mediated inflammation.

As the first approved IL-1 inhibitor (2001), anakinra remains a basic drug for the treatment of refractory RA and neonatal multi-system inflammatory diseases through inhibiting the binding of IL-1α/β to receptors [5]. Approved in 2008, rilonacept effectively neutralizes IL-1α and IL-1β, showing distinctive efficacy in treating CAPS and recurrent pericarditis [6]. Approved in 2009, canakinumab has shown therapeutic efficacy by selectively neutralizing IL-1β in various diseases, such as CAPS, tumor necrosis factor receptor-associated periodic syndrome (TRAPS), and reducing atherosclerotic cardiovascular risk in certain populations [7]. Despite their efficacy, IL-1 inhibitors carry significant risks, including severe infections and hematologic abnormalities, primarily due to systemic immunosuppression [8]. Moreover, current safety profiles primarily derive from clinical trials and small observational studies, which limit long-term risk assessment and broader applicability [9].

The FDA Adverse Event Reporting System (FAERS) is a publicly accessible pharmacovigilance database containing over 20 million spontaneous adverse event (AE) reports. It is essential for post-marketing surveillance, facilitating signal detection via disproportionality analysis and stratification by patient demographics or drug interactions [10,11]. Therefore, this study systematically mined AEs for IL-1 inhibitors using FAERS data to quantify AE reporting frequencies and signal strengths, identify risk signals requiring clinical attention, and compare safety profiles among IL-1 inhibitors, further guiding risk mitigation strategies and informing therapeutic decision-making in chronic inflammatory diseases.

## 2. Results

### 2.1. Basic Information of AEs

From Q1 2004 to Q3 2024, 3,549,253 duplicate reports were removed, resulting in a final analytical cohort of 18,289,374 unique cases. From this cohort, we extracted 17,670 cases that listed an IL-1 inhibitor as a primary suspect (PS), comprising 7927 AEs for anakinra, 9277 AEs for canakinumab, and 466 AEs for rilonacept. Table 1 presents the baseline characteristics of these AEs. In terms of gender, 57.7% of IL-1 inhibitor reports, 64.3% of anakinra reports, 51.9% of canakinumab reports, and 58.8% of rilonacept reports were from the female population. In terms of age, the rate for IL-1 inhibitors was 17.8% (<18), 24.6% (18–65), and 8% (>65). The distribution of age for anakinra reports was 12.7% (<18), 30.5% (18–65), 9.6% (>65). For canakinumab, the rate was 23.0% (<18), 18.5% (18–65), and 6.1% (>65). For rilonacept, most reports occurred in individuals aged between 18 and 65, accounting for 44.2%. Regarding reporter occupation, consumer reports accounted for a considerable proportion across different IL-1 inhibitors (IL-1 inhibitors: 55.7%, anakinra: 66.1%, canakinumab: 48.6%), whereas health professionals were the predominant reporters for rilonacept (68.7%). Although consumer reports broaden information sources, consumers’ lack of professional medical knowledge may limit the accuracy and detail of adverse reaction information. In contrast, health professionals, leveraging their expertise, can furnish more precise and comprehensive AE-related data.

**Figure 1:**
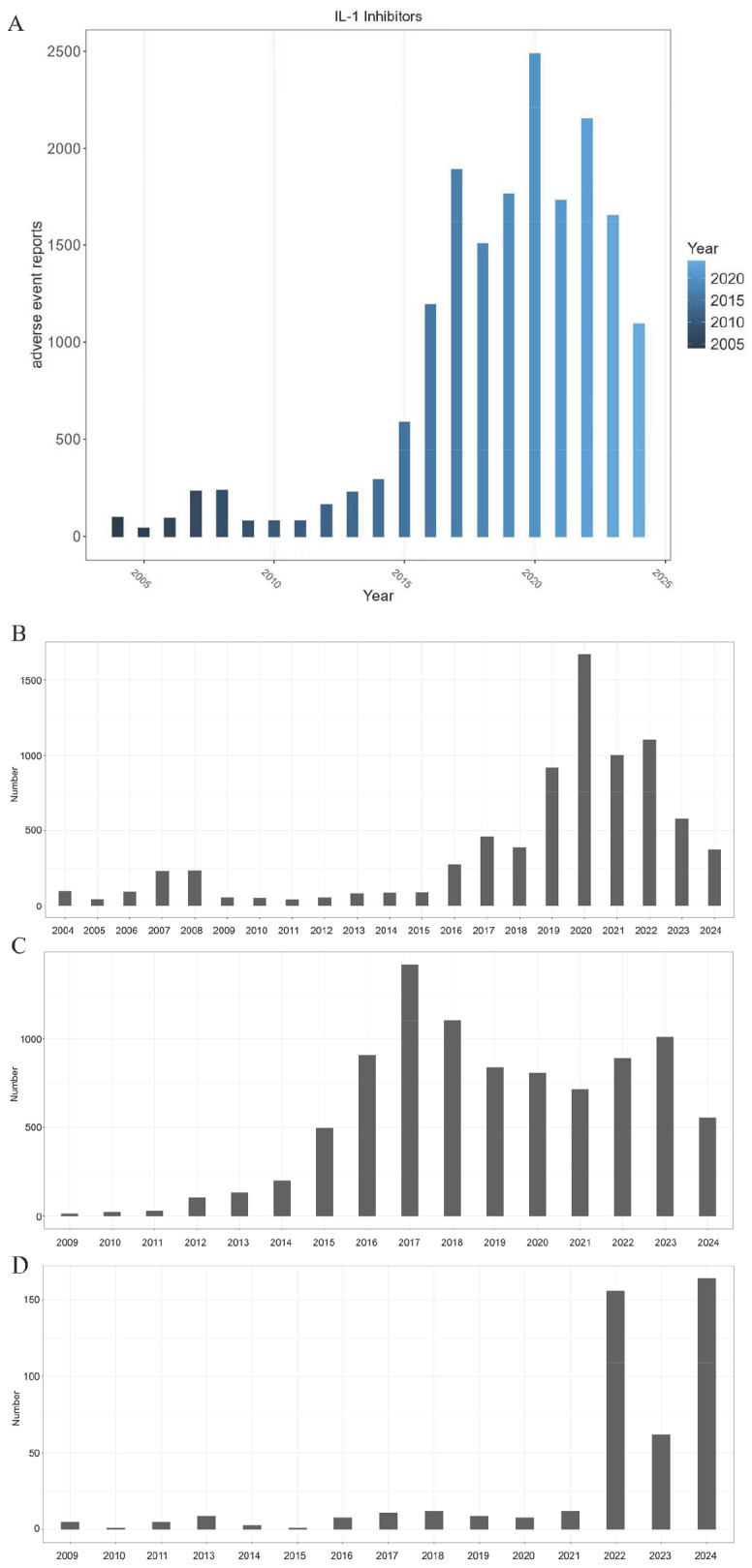
The annual distribution of IL-1 inhibitor-related reported cases. (**A**) Overall reporting trends for the IL-1 inhibitor class. (**B**) Anakinra. (**C**) Canakinumab. (**D**) Rilonacept. The horizontal axis represents reporting years (2004–2024), while the vertical axis shows the number of adverse event cases. Bar height corresponds to the annual case volume, illustrating temporal variations in reporting frequency.

Figure 1 depicts the yearly distribution of AEs associated with IL-1 inhibitors. The peak of IL-1 inhibitors was in 2020 with 2486 (Figure 1A). Anakinra-related reports peaked in 2020 with 1670 (Figure 1B). Canakinumab-related reports peaked in 2017 with 1422 (Figure 1C). For rilonacept, there were two peaks in the number of reports, one in 2022 (*n* = 156) and the other in 2024 (*n* = 164) (Figure 1D). These temporal trends in AE reporting closely reflect key phases of clinical practice, including the repurposing of agents during the COVID-19 pandemic, the expansion into new cardiovascular indications, and post-approval surveillance for recently approved uses, thereby providing real-world insights into the evolving safety profiles of these therapeutics.

### 2.2. Disproportionality Analysis

**Figure 2:**
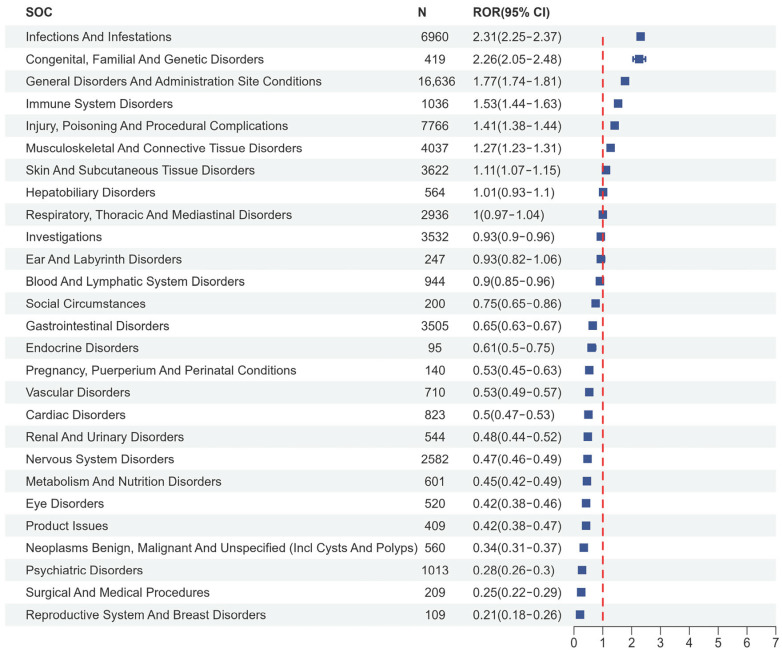
Forest map of system organ classes (SOCs) induced by IL-1 inhibitors. For each SOC, the blue box displays the ROR value and its 95% confidence interval (CI), alongside the number of adverse event reports (N). The vertical dashed line indicates no association (ROR = 1). SOCs with a 95% CI not crossing this line represent statistically significant signals.

At the system organ class (SOC) level, the distribution of AEs induced by IL-1 inhibitors as PS was shown in Figure 2. Results demonstrated that 27 SOCs associated with IL-1 inhibitor-related AEs were identified. General disorders and administration site conditions accounted for the highest percentage of SOCs at 27.4%, followed by injury, poisoning, and procedural complications at 12.79%, and infections and infestations at 11.46%. Infections and infestations (reporting odds ratio (ROR): 2.31, 95% CI: 2.25–2.37) and congenital, familial, and genetic disorders (ROR: 2.26, 95% CI: 2.05–2.48) were two SOCs meeting all four criteria simultaneously (see Supplementary Table S1). Moreover, there were 27 SOCs for anakinra, 27 SOCs for canakinumab, and 26 SOCs for rilonacept, and the top 3 SOCs were consistent with IL-1 inhibitors (Supplementary Table S1). anakinra and rilonacept did not show positive signals at the SOC level, whereas canakinumab exhibited positive signals for infections and infestations (ROR: 2.58, 95% CI 2.49–2.67) and congenital, familial, and genetic disorders (ROR: 3.89, 95% CI 3.49–4.33).

Additionally, the disproportionality analysis identified a total of 263, 158, 231, and 40 significant preferred terms (PTs) for the IL-1 inhibitor classes, anakinra, canakinumab, and rilonacept, respectively. Table S2 provides the complete dataset for these signals, encompassing the raw cell counts (a, b, c, d) for all PTs that were significant across all four algorithms. Table 2 displays the top 20 PTs for each drug, ranked by case frequency, to improve visualization (pericarditis in cardiac disorders and Still’s Disease signals were excluded as indications). Among these PTs, injection-site reaction (including pyrexia (ROR: 5.27, 95% CI = 5.03–5.53), injection site pain (ROR: 3.8, 95% CI = 3.58–4.04), injection site erythema (ROR: 6.09, 95% CI = 5.67–6.55)), various infections (including nasopharyngitis (ROR: 2.31, 95% CI = 2.1–2.54), upper respiratory tract infection (ROR: 2.6, 95% CI = 2.17–3.11), gastroenteritis (ROR: 9.23, 95% CI = 3.83–22.29)), and so on were aligned with the warnings and precautions stated in the drug labels. Moreover, in our findings, we identified that additional adverse reactions were uncommon in the prescribing information. For instance, cardiac disorders were identified in anakinra (including postural orthostatic tachycardia syndrome (POTS), ROR: 7.39, 95% CI = 2.77–19.74) and rilonacept (including pulmonary valve incompetence (PVI), ROR: 88.13, 95% CI = 28.35–273.97). For canakinumab, endocrine disorders (including secondary adrenocortical insufficiency, ROR: 6.3, 95% CI = 2.36–16.8; cushingoid, ROR: 7.61, 95% CI = 4.21–13.77) and eye disorders (papilloedema, ROR: 5.2, 95% CI = 2.88–9.4) were observed.

Subgroup analyses by gender and age displayed different patterns (Supplementary Tables S3–S5). Excepting common AEs, IL-1 inhibitor therapy was associated with several clinically important sex- and age-specific risks. In male patients, significant signals were observed for psychiatric disorders (autism spectrum disorder, ROR: 3.72, 95% CI = 1.93–7.17), ear and labyrinth disorders (neurosensory deafness, ROR: 4.56, 95% CI = 2.04–10.16), and eye disorders (conjunctival hyperaemia, ROR: 4.1, 95% CI = 1.84–9.14). A strong signal for renal amyloidosis was identified predominantly in females (ROR: 45.78, 95% CI = 20.1–104.28) and the 18–65 age group (ROR: 63.79, 95% CI = 27.91–145.79). Notably, pediatric-specific risks (0–18 years) were identified. A significant signal for secondary adrenocortical insufficiency was found for the IL-1 inhibitor class (ROR: 7.09, 95% CI = 2.6–19.35) and specifically for canakinumab (ROR: 7.8, 95% CI = 2.46–24.7).

To address potential reporting bias arising from heterogeneous reporter types (e.g., over-reporting of injection-site reactions by consumers), we performed a subgroup disproportionality analysis stratified by consumer (CN) and healthcare professional (HP/MD) reports. The results demonstrated consistent safety signals across reporter subgroups for key AEs. For instance, the signal for pyrexia was significant in both CN and HP/MD reports for canakinumab (HP/MD ROR: 6.04, 95% CI = 5.47–6.66; CN ROR: 11.79, 95% CI = 10.81–12.87) and anakinra (HP/MD ROR: 2.62, 95% CI = 2.14–3.21; CN ROR: 3.21, 95% CI = 2.87–3.6). Similarly, injection site erythema was a robust signal in both subgroups for anakinra (HP/MD ROR: 4.68, 95% CI = 3.59–6.10; CN ROR: 11.37, 95% CI = 10.44–12.39), and rilonacept (HP/MD ROR: 8.64, 95% CI = 3.21–23.27; CN ROR: 14.73, 95% CI = 8.07–26.87). As anticipated, ROR point estimates for subjective and local injection-site reactions were higher in consumer reports. However, the same signals were unequivocally positive and statistically significant in the HP/MD reports, confirming their robustness. This consistency across reporter subgroups reinforces the primary safety findings for IL-1 inhibitors. Detailed results are provided in Table S5.

### 2.3. Time to Onset of IL-1 Inhibitor-Related AEs

**Figure 3:**
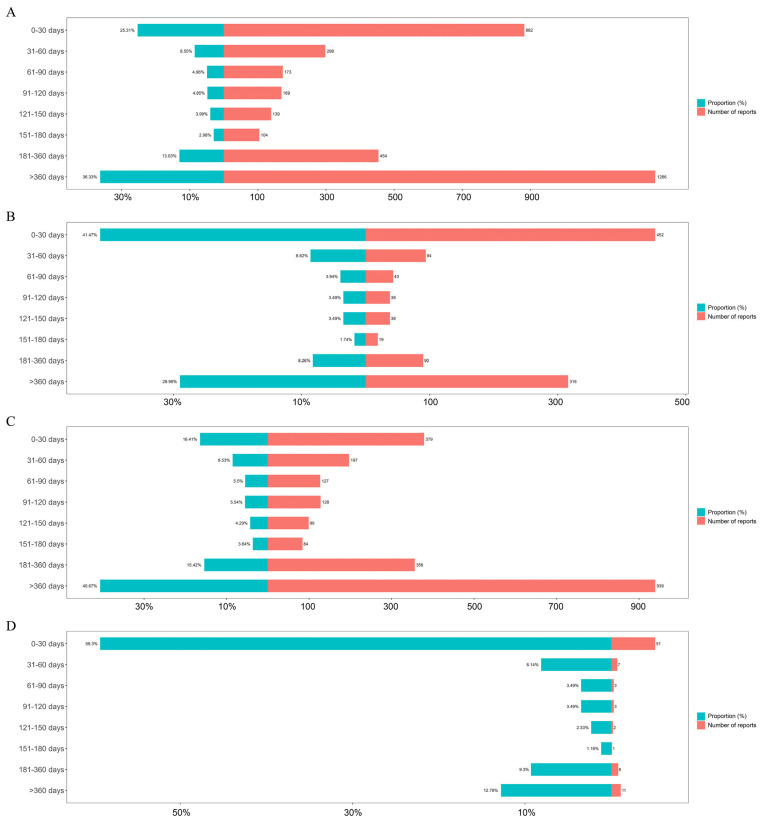
Onset time distribution of IL-1 inhibitor-related AEs. (**A**) IL-1 inhibitors. (**B**) anakinra. (**C**) canakinumab. (**D**) rilonacept. Each panel displays the proportion of cases (left vertical axis, expressed as a percentage) and the absolute number of reported cases (right vertical axis, expressed as a count) across defined time intervals (horizontal axis). Time-to-onset is categorized into sequential periods from 0 to 30 days to over 300 days post-treatment initiation, illustrating the temporal profile of reported AEs for each therapeutic agent.

**Figure 4:**
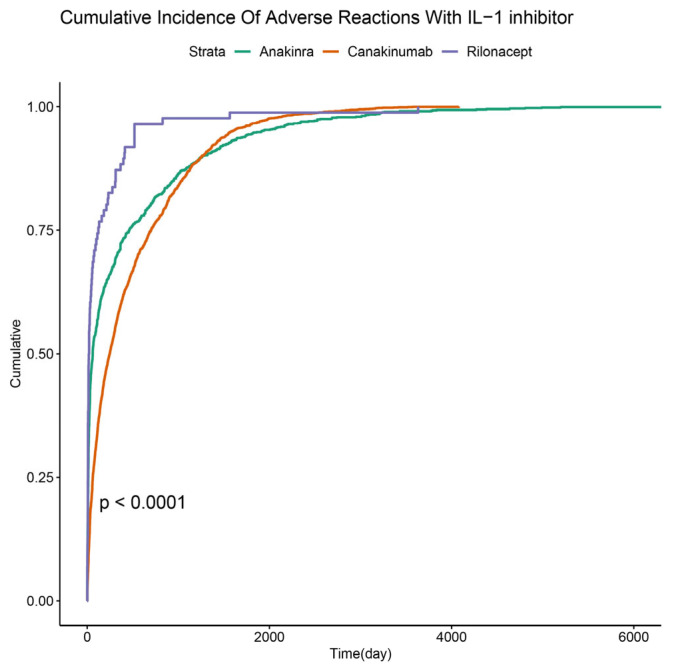
Cumulative incidence of AEs stratified by three IL-1 inhibitors (Gray’s test: *p <* 0.0001). The vertical dashed line indicates the daytime point.

We also analyzed the onset time of IL-1 inhibitor-related AEs (Figure 3). The findings indicated that most cases (36.33%) emerged after more than a year (>360 days) of IL-1 inhibitor treatment, with the next highest occurrence (25.31%) in the first month (Figure 3A). For populations with anakinra treatment, most cases occurred in the first month (41.47%) (Figure 3B). After one year of canakinumab treatment, 40.67% of patients experienced AEs, with 16.41% reporting AEs within the first 30 days (Figure 3C). For rilonacept, AEs were reported in 59.3% of patients within 30 days and 12.79% after one year of treatment (Figure 3D). To further characterize these risks over time, we performed a cumulative incidence analysis, which confirmed a significantly different temporal distribution of AE risk among the three drugs (Gray’s test, *p <* 0.0001; Figure 4).

## 3. Discussion

Utilizing FAERS database (2004–2024; 17,670 IL-1 inhibitor-related AE reports), our analysis not only confirmed established drug-specific safety profiles—such as injection site reactions with anakinra and gastrointestinal/respiratory disorders with canakinumab—but also provided a systematic evaluation of IL-1 inhibitors safety as a whole class (alongside head-to-head comparisons among anakinra, canakinumab, rilonacept), identifying novel drug-specific AEs. Specifically, compared with the single-drug study on rilonacept (419 reports, 2021–2024) [12]—which primarily revealed causal effects of rilonacept on allergic urticaria, rash, and myocarditis—our study included rilonacept-related reports spanning 20 years and further identified a novel signal for cardiac disorders: PVI, which avoids biases associated with short-term follow-up and captures rare risk trends that short-duration studies cannot detect. Meanwhile, the inclusion of all three IL-1 inhibitors (as well as whole IL-1 inhibitors class) in our analysis—unlike prior two-drug investigations that focused solely on anakinra and canakinumab (2004–2023)—enabled the identification of inter-drug disparities in infection risk that remained undetected in narrower-scope studies [13]: canakinumab demonstrated a markedly higher disproportionality signal for serious infections (e.g., pneumonia) relative to anakinra and rilonacept, underscoring a distinct infection risk profile among IL-1 inhibitors. While other adverse reactions not commonly noted in prescribing information—specifically cardiac disorders—were identified in anakinra (POTS) and rilonacept (PVI). Furthermore, subgroup analysis of the pediatric population revealed a unique risk of secondary adrenocortical insufficiency associated with canakinumab. This endocrine risk profile expands upon the previously identified risks of gastrointestinal and respiratory disorders induced by canakinumab in minors. These findings provided actionable evidence for optimizing therapeutic monitoring of IL-1 inhibitors in clinical practice.

### 3.1. Reporting Trends in the Context of Drug Lifecycles

The annual distribution of AEs associated with IL-1 inhibitors revealed two notable trends regarding anakinra, which not only reflect its clinical application dynamics but also provide critical real-world evidence for optimizing medication safety management. First, 2020 emerged as the peak year for both overall IL-1 inhibitor-related AEs and anakinra-specific occurrences, a pattern that aligns with the widespread off-label use of IL-1 inhibitors—including anakinra—for the treatment of coronavirus disease 2019 (COVID-19) during this period [14,15,16], especially mitigating the “cytokine storm” in critically ill COVID-19 patients [17]. This temporal correlation reminds clinicians to balance the therapeutic benefits of anakinra (and other IL-1 inhibitors) in emergency scenarios like severe COVID-19 with close monitoring for potential AEs. Second, the proportion of anakinra reports significantly decreased after 2010; this trend is linked to the introduction of alternative therapies with fewer side effects, such as interleukin-6 receptor antagonists (e.g., tocilizumab) and JAK inhibitors (e.g., tofacitinib)—which have partially diminished anakinra’s market share in clinical practice. Furthermore, incidences associated with canakinumab peaked in 2017, with its reports constituting the majority (58%) of IL-1 inhibitor-related reports, reflecting its growing clinical applications, notably its 2017 approval for inflammation management in atherosclerotic cardiovascular disease (ASCVD) patients [1]. This trend not only confirms canakinumab’s growing adoption in ASCVD care but also underscores the need for long-term AE surveillance in this patient population. Notably, rilonacept-related AE reports showed explosive growth (a 120% increase between 2021 and 2023) following its 2021 approval for recurrent pericarditis, which directly reflected the growth of clinical demand caused by this indication [18]. Clinically, while the rise in AEs requires vigilant monitoring, it also highlights the importance of AE surveillance for newly approved indications, which is critical to refining dosing strategies and identifying population-specific safety signals, underscoring the dynamic interplay between drug lifecycle phases and AE surveillance priorities.

Sex disparities were observed among the reports, with female patients accounting for 64% of cases—this distribution aligns with the known female predominance in IL-1-mediated conditions (e.g., adult-onset Still’s disease), while pediatric underrepresentation (19% of reports) may stem from limited off-label prescribing in children. However, given the FAERS database’s inherent limitations (e.g., potential underreporting, selective reporting of specific demographics or AEs), the observed gender- and age-related reporting trends should be interpreted cautiously, as they may not accurately represent true AE incidence across populations nor provide definitive evidence of demographic-specific risks.

More than half of the reports were reported by consumers (55.7%), a phenomenon closely linked to the US FDA’s 2013 reporting system reform [19], which introduced a simplified consumer-centric reporting form (FDA 3500B) and optimized the electronic submission process—measures that lowered consumer reporting barriers, boosted report quantity, and expanded adverse reaction monitoring scale. However, it is important to acknowledge key limitations of expanded consumer participation: the inclusion of multiple reporting sources elevates the likelihood of redundant entries—with deduplication in the present analysis relying solely on case IDs, potentially introducing data biases—and the surge in consumer reports may also compromise reliability (e.g., inaccurate identification of AEs or incomplete details like drug dosage). In contrast, health professionals—who submitted 68.7% of reports for rilonacept—are able to provide more clinically structured and detailed information due to their systematic medical expertise. The integration of reports from both consumers and healthcare professionals is a well-established practice in pharmacovigilance, aimed at capturing a broader spectrum of safety information [20,21,22].

### 3.2. Mechanistic Correlates of Signal Disproportionality of IL-1 Inhibitors: Risk of Infection

The robust disproportionality signals observed for the SOC “infections and infestations” (ROR: 1.37, 95% CI = 1.13–1.65) directly stem from the dual role of IL-1β in orchestrating both innate immune defense against pathogens and regulation of autoinflammatory cascades [7]. Physiologically, IL-1β is a key mediator of the host response to microbial invasion, activating neutrophil recruitment, cytokine secretion, and acute-phase protein production to clear pathogens [23]. However, its overproduction drives pathological inflammation in autoinflammatory disorders, necessitating therapeutic inhibition [24]. This duality creates a therapeutic paradox: while IL-1 inhibitors effectively dampen aberrant inflammation, they concomitantly blunt protective immune responses, increasing susceptibility to infections [25].

At the PT level, specific infections are aligned with this mechanism. Nasopharyngitis (ROR 2.31, 95% CI: 2.1–2.54) and upper respiratory tract infections (ROR 2.6, 95% CI: 2.17–3.11) were notable, indicating compromised mucosal immunity, a key defense mechanism where IL-1β is essential for pathogen clearance [26,27]. Notably, differences in the ROR values among different drugs further highlight the mechanistic nuances: canakinumab, a selective IL-1β neutralizer, reported data on pneumonia with an ROR of 2.3 (95% CI: 2.07–2.56), whereas neither anakinra nor rilonacept reported an ROR for this event. This may relate to canakinumab’s longer half-life (26 days) and more sustained IL-1β inhibition, whereas anakinra’s short half-life (4–6 h) and rilonacept’s dual IL-1α/β targeting might preserve partial immune competence [28,29]. These data align with prior meta-analyses highlighting canakinumab’s elevated infection risk in autoimmune populations [30].

Moreover, the SOC “congenital, familial and genetic disorders” (ROR: 2.26, 95% CI = 2.05–2.48) also warrants attention, with PTs such as familial Mediterranean fever exacerbations (ROR: 1047.69, 95% CI = 781.25–1405) showing strong signals. IL-1β is central to the pathogenesis of hereditary autoinflammatory syndromes, and while inhibition is therapeutic, abrupt suppression may disrupt homeostatic immune signaling in genetically predisposed individuals, potentially unmasking or exacerbating underlying genetic susceptibilities [24,31,32].

Beyond expected signals, several novel AEs emerged, extending current safety profiles. For anakinra, POTS (ROR: 7.39, 95% CI = 2.77–19.74) was identified as a potential association not previously documented in the literature. To further evaluate this signal, we performed a detailed narrative review of individual case reports ( Table S6). A notable female predominance (3 of 4 cases) was observed, aligning with the known epidemiology of POTS, thereby enhancing the biological plausibility of this signal [33]. All reports originated with consumers, suggesting they may represent less severe cases that did not result in hospitalization, yet they highlight a meaningful real-world patient experience. This signal may be attributed to adverse effects induced by the excessive use of anakinra for the treatment of pericarditis. Consequently, future real-world studies should incorporate close monitoring of anakinra dosage to further clarify this potential risk. Similarly, a strong signal was detected for PVI with rilonacept (ROR: 88.13; 95% CI = 28.35–273.97). Case narrative review revealed a remarkably consistent pattern: all three cases involved 5-year-old girls being treated for Juvenile Arthritis, and all were serious, physician-reported events. This homogeneity makes a random reporting artifact unlikely and suggests a potential specific risk in this susceptible demographic. However, the very low case number and clustering within a narrow demographic and geographic scope necessitate cautious interpretation, as this could be influenced by regional prescribing practices or other surveillance artifacts. Therefore, while this signal warrants clinical awareness, its confirmation requires further investigation. This finding stands in contrast to evidence supporting the safe use of anakinra in patients with pulmonary arterial hypertension (PAH) [34,35]. Given the current lack of evidence linking rilonacept to pulmonary valve function changes, it seems prudent to recommend that patients with significant pre-existing pulmonary valve regurgitation or pulmonary hypertension should receive a baseline cardiac work-up and infection-risk assessment before starting an IL-1 inhibitor, and those with recurrent pericarditis who develop right-heart failure or elevated pulmonary artery pressure should have pulmonary pressure measured to exclude secondary PVI. For canakinumab, endocrine disorders such as secondary adrenocortical insufficiency (ROR: 6.3, 95% CI = 2.36–16.8) and cushingoid (ROR: 7.61, 95% CI = 4.21–13.77) were notable. While not previously emphasized in labeling, these may arise from IL-1β’s role in hypothalamic–pituitary–adrenal axis regulation; sustained inhibition could dysregulate cortisol synthesis, particularly in pediatric populations with developing endocrine systems [36]. Notably, although these AEs had lower absolute report counts, their elevated RORs suggest non-random associations that merit prospective monitoring.

Subgroup analyses by age, sex, and reporter type revealed distinct patterns in the safety profile of IL-1 inhibitors, with implications for both mechanistic understanding and the robustness of the findings. In pediatric populations (<18 years), secondary adrenocortical insufficiency (ROR: 7.09, 95% CI = 2.6–19.35) was more prevalent, likely due to immature immune-endocrine crosstalk—IL-1β is a critical mediator of stress-induced cortisol release in children, and its inhibition may disrupt this axis [37,38,39]. In male patients, autism spectrum disorder (ROR: 3.72, 95% CI = 1.93–7.17) and neurosensory deafness (ROR: 4.56, 95% CI = 2.04–10.16) were observed. Preclinical studies suggest IL-1 signaling modulates neurodevelopmental pathways, and sex-specific differences in IL-1 receptor expression in the central nervous system may render males more susceptible to perturbations [40,41]. Conversely, renal amyloidosis was more frequent in females (ROR: 45.78, 95% CI = 20.1–104.28) and the 18–65 (ROR: 63.79, 95% CI = 27.91–145.79) age group. However, existing literature reports that renal amyloidosis is more common in men [42]. Additionally, there is no direct evidence to prove a correlation between its incidence and severity with IL-1β and estrogen [43]. The reason for this result may be related to gender bias in the data, and its reliability needs to be confirmed through long-term clinical observation. Critically, the subgroup analysis by reporter type demonstrated the robustness of the core safety signals. Although consumer reports showed higher ROR magnitudes for local and subjective AEs (e.g., injection site reactions), these signals remained statistically significant and consistent in reports from healthcare professionals. This concordance across different reporter types strengthens the validity of the primary safety findings and mitigates concerns about reporting bias.

### 3.3. Limitations and Future Directions

Our study has several important limitations inherent to its design and the use of spontaneous reporting system data [44,45,46]. First, and most critically, the findings from disproportionality analyses are susceptible to various biases and do not establish causality. Key among these is confounding by indication. This is particularly relevant for the strong infection signal associated with canakinumab, as this drug is prescribed for autoinflammatory syndromes (e.g., CAPS, sJIA), which intrinsically predispose patients to a higher risk of infections due to the underlying immune dysregulation [47]. Therefore, the strong disproportionality signal observed (ROR 3.89) likely reflects this confounding effect and cannot be solely attributed to the drug itself. Similarly, other identified signals may be influenced by the underlying diseases being treated rather than the drug itself. Our analysis, which compares each IL-1 inhibitor against all other drugs in the database, cannot adequately adjust for this fundamental source of confounding, nor for confounding introduced by concomitant medications. Additional biases, such as channeling bias (where drugs are prescribed to specific patient populations), protopathic bias (where early symptoms of a disease are mistakenly attributed to a drug), and notoriety bias (increased reporting following increased clinical attention, as potentially seen with anakinra during the COVID-19 pandemic), could also influence the observed signals.

Second, the structure of the FAERS database presents analytical constraints. The database lacks a dedicated follow-up flag and accessible longitudinal linkage information, which precluded a sensitivity analysis to assess the impact of follow-up reports on signal robustness. While our deduplication process prioritized the most recent report to minimize residual duplication, we cannot fully rule out its potential influence. Furthermore, the database does not routinely provide detailed clinical information on patient comorbidities, disease severity, or precise temporal sequences, which limits our ability to perform more sophisticated causal inference analyses.

Third, signals for rare events, such as POTS associated with anakinra and PVI associated with rilonacept, must be interpreted with extreme caution. The individual case reports for these signals are based on a very small number of cases and could be influenced by regional prescribing patterns or surveillance artifacts. They should be considered as preliminary hypotheses that require validation through larger, targeted pharmacoepidemiologic studies capable of robustly controlling for confounders.

In light of these limitations, our results should be viewed as hypothesis-generating. They highlight potential safety concerns that warrant further investigation in studies designed to establish causality, such as those using electronic health records with longitudinal data or claims databases. Preclinical models exploring IL-1 isoform-specific toxicity could further clarify observed subgroup disparities.

## 4. Materials and Methods

### 4.1. Study Design and Data Sources

**Figure 5:**
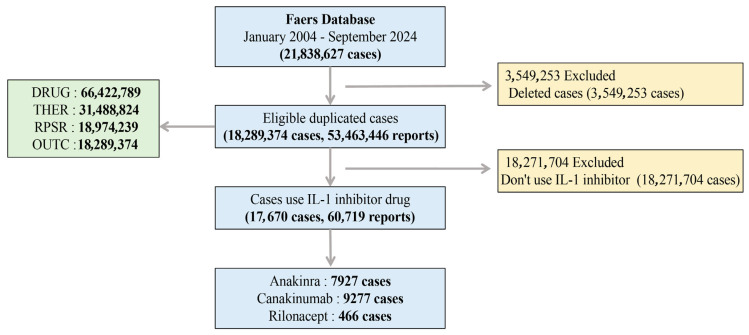
Flowchart of the screening process for IL-1 inhibitor-related AEs.

Figure 5 outlines the detailed data extraction and case selection process from the FAERS database (https://www.fda.gov/drugs/drug-approvals-and-databases/fda-adverse-event-reporting-system-faers-database (accessed on 9 November 2025)). In this retrospective study, AEs associated with IL-1 inhibitors (anakinra, canakinumab, and rilonacept) were analyzed using FAERS data from Q1 2004 to Q3 2024. The FAERS database, updated quarterly, consists of seven datasets: demographic and administrative information (DEMO), drug information (DRUG), adverse drug reaction information (REAC), patient outcomes (OUCT), reported sources (RPSR), drug therapy start and end dates (THER), and drug administration indications (INDI) [12]. The RxNorm system was utilized to standardize drug names, while MedDRA was used to standardize the SOC and PT for collected AEs. The FDA advises removing duplicate data prior to conducting statistical analysis. When CASEID matched, the most recent FDA_DTs were chosen. If CASEID and FDA_DTs matched, the higher PRIMARYID was prioritized [48]. FAERS reports, with duplicates removed, were analyzed for cases listing ‘anakinra,’ ‘canakinumab,’ and ‘rilonacept’ as the PS.

### 4.2. Statistical Analysis

Disproportionality analysis was conducted to detect signals by comparing the proportion of target events linked to the target drug with those linked to all other drugs (Table 3). This study employed a combination of two frequentist methods (ROR; Proportional Reporting Ratio, PRR) and two Bayesian approaches (Bayesian Confidence Propagation Neural Network, BCPNN; Multi-item Gamma Poisson Shrinker, MGPS). As detailed in Table 4, each algorithm possesses a unique calculation process, detection threshold, and evaluation criteria. In our study, drugs that met the criteria of all four methods simultaneously were positive signals, illustrating a significant correlation between the drugs and the events. This multi-method strategy was adopted to leverage their complementary strengths, thereby enhancing the robustness of our findings and mitigating the inherent limitations of any single method [49,50]. Moreover, we conducted subgroup analyses to investigate the connections between IL-1 inhibitors and adverse effects by different age groups (<18 (child), 18–65 (middle age), >65 (elder)), gender (male and female), and reporter type (Consumer [CN] vs. Healthcare Professional [HP/MD]). Additionally, onset times of IL-1 inhibitor-related PTs were analyzed. Statistical analyses were conducted using R software (version 4.2.1).

## 5. Conclusions

In conclusion, this study, leveraging the FAERS database, yielded three key, specific findings that advance the understanding of IL-1 inhibitor safety in real-world settings: first, it systematically compared the safety profiles of three clinically relevant IL-1 inhibitors (anakinra, canakinumab, and rilonacept), clarifying both shared and distinct safety signals among them; second, it confirmed the well-documented infection risk associated with IL-1 inhibition, providing real-world evidence to reinforce this established safety concern; and third, it identified drug-specific safety characteristics—specifically, anakinra may be associated with POTS, while rilonacept may be linked to PVI. Notably, these newly identified safety signals are currently derived solely from this retrospective pharmacovigilance analysis and lack support from independent literature reports or prospective validation. Nevertheless, these findings provide valuable preliminary real-world insights into the safety profiles of the IL-1 inhibitors, laying a foundational reference for exploring individualized clinical monitoring strategies to address agent-specific risks.

## Figures and Tables

**Table 1:** Clinical characteristics of patients with AEs caused by IL-1 inhibitors.

Characteristics	Variable	IL-1 Inhibitors	Anakinra	Canakinumab	Rilonacept
Overall		(*N* = 17,670)	(*N* = 7927)	(*N* = 9277)	(*N* = 466)
Gender	Female	10,189 (57.7%)	5100 (64.3%)	4815 (51.9%)	274 (58.8%)
	Male	6210 (35.1%)	2628 (33.2%)	3461 (37.3%)	121 (26.0%)
	Missing	1271 (7.2%)	199 (2.5%)	1001 (10.8%)	71 (15.2%)
Age	<18	3153 (17.8%)	1009 (12.7%)	2133 (23.0%)	11 (2.4%)
	18–65	4341 (24.6%)	2415 (30.5%)	1720 (18.5%)	206 (44.2%)
	>65	1401 (8%)	766 (9.6%)	561 (6.1%)	74 (15.9%)
	Missing	8775 (49.7%)	3737 (47.1%)	4863 (52.4%)	175 (37.6%)
Reporter’s occupation	Consumer (CN)	9841 (55.7%)	5243 (66.1%)	4510 (48.6%)	88 (18.9%)
	Health-professional (HP)	2302 (13.0%)	426 (5.4%)	1556 (16.8%)	320 (68.7%)
	Lawyer (LW)	2 (0.0%)	2 (0.0%)		
	Physician (MD)	3535 (20.0%)	1269 (16.0%)	2226 (24.0%)	40 (8.6%)
	Other health professional (OT)	1251 (7.1%)	435 (5.5%)	811 (8.7%)	5 (1.1%)
	Pharmacist (PH)	299 (1.7%)	186 (2.3%)	104 (1.1%)	9 (1.9%)
	Registered Nurse (RN)	12 (0.1%)	8 (0.1%)	3 (0.0%)	1 (0.2%)
	Missing	428 (2.4%)	358 (4.5%)	67 (0.7%)	3 (0.6%)

**Table 2:** Top 20 signal strength of IL-1 inhibitor-related reports in the FAERS database at the preferred term (PT) level.

Name	SOC Name	PT	*n*	ROR	PRR	EBGM (EBGM05)	IC (IC025)
				(95% Cl)			
IL-1 inhibitors	General Disorders and Administration Site Conditions	Pyrexia	1812	5.27 (5.03–5.53)	5.14 (6050.1)	5.12 (4.92)	2.36 (2.29)
		Condition Aggravated	1098	3.84 (3.61–4.07)	3.78 (2250.95)	3.77 (3.59)	1.92 (1.83)
		Injection Site Pain	1080	3.8 (3.58–4.04)	3.75 (2183.13)	3.74 (3.56)	1.9 (1.82)
		Injection Site Erythema	744	6.09 (5.67–6.55)	6.03 (3108.52)	6 (5.64)	2.58 (2.48)
		Injection Site Pruritus	542	8.31 (7.63–9.04)	8.24 (3420.51)	8.17 (7.61)	3.03 (2.91)
		Injection Site Reaction	486	7.19 (6.57–7.86)	7.14 (2548.11)	7.09 (6.58)	2.83 (2.69)
		Illness	423	5.32 (4.83–5.86)	5.29 (1465.15)	5.27 (4.86)	2.4 (2.26)
		Injection Site Swelling	346	4.75 (4.28–5.28)	4.73 (1014.18)	4.71 (4.31)	2.24 (2.08)
		Injection Site Rash	340	11.93 (10.72–13.28)	11.87 (3340.06)	11.72 (10.72)	3.55 (3.39)
		Injection Site Urticaria	329	14.43 (12.94–16.1)	14.36 (4025.36)	14.15 (12.91)	3.82 (3.66)
		Injection Site Bruising	316	4.16 (3.72–4.64)	4.14 (749.98)	4.13 (3.76)	2.04 (1.88)
	Infections and Infestations	COVID-19	698	3.92 (3.64–4.23)	3.89 (1495.24)	3.88 (3.64)	1.95 (1.84)
		Infection	462	3.29 (3–3.6)	3.27 (726.81)	3.26 (3.02)	1.71 (1.57)
		Nasopharyngitis	419	2.31 (2.1–2.54)	2.3 (307.88)	2.3 (2.12)	1.2 (1.06)
		Influenza	318	3 (2.69–3.35)	2.99 (420.33)	2.98 (2.72)	1.58 (1.41)
	Injury, Poisoning, and Procedural Complications	Off-Label Use	2793	3.56 (3.43–3.7)	3.44 (4892.7)	3.44 (3.33)	1.78 (1.72)
		Product Dose Omission Issue	1028	4.66 (4.38–4.95)	4.6 (2887.51)	4.58 (4.35)	2.19 (2.1)
		Inappropriate Schedule of Product Administration	941	6.22 (5.83–6.64)	6.14 (4030.46)	6.1 (5.78)	2.61 (2.51)
		Incorrect Dose Administered	523	2.6 (2.38–2.83)	2.58 (508.05)	2.58 (2.4)	1.37 (1.24)
	Skin and Subcutaneous Tissue Disorders	Urticaria	406	2.51 (2.28–2.77)	2.5 (365.06)	2.49 (2.3)	1.32 (1.18)
anakinra	General Disorders and Administration Site Conditions	Injection Site Pain	928	6.47 (6.06–6.91)	6.31 (4151.35)	6.29 (5.96)	2.65 (2.56)
		Injection Site Erythema	641	10.36 (9.58–11.2)	10.16 (5275.45)	10.11 (9.47)	3.34 (3.22)
		Pyrexia	503	2.81 (2.58–3.07)	2.78 (577.24)	2.78 (2.58)	1.48 (1.35)
		Injection Site Pruritus	493	14.88 (13.61–16.27)	14.66 (6229.43)	14.55 (13.5)	3.86 (3.73)
		Condition Aggravated	440	2.99 (2.72–3.28)	2.96 (573.22)	2.96 (2.73)	1.56 (1.43)
		Injection Site Reaction	421	12.25 (11.12–13.49)	12.09 (4258.69)	12.02 (11.08)	3.59 (3.44)
		Injection Site Urticaria	307	26.45 (23.62–29.63)	26.2 (7332.53)	25.82 (23.49)	4.69 (4.52)
		Injection Site Swelling	295	7.96 (7.09–8.93)	7.89 (1769.21)	7.86 (7.14)	2.97 (2.81)
		Injection Site Rash	293	20.16 (17.96–22.64)	19.98 (5224.76)	19.76 (17.94)	4.3 (4.13)
		Injection Site Bruising	283	7.31 (6.5–8.22)	7.25 (1521.31)	7.23 (6.55)	2.85 (2.68)
		Illness	172	4.21 (3.63–4.9)	4.2 (418.25)	4.19 (3.69)	2.07 (1.85)
	Infections and Infestations	COVID-19	420	4.62 (4.2–5.09)	4.57 (1172.18)	4.56 (4.21)	2.19 (2.05)
		Infection	286	3.98 (3.55–4.48)	3.96 (631.74)	3.95 (3.58)	1.98 (1.81)
		Sinusitis	159	3 (2.57–3.51)	2.99 (211.05)	2.99 (2.62)	1.58 (1.35)
	Injury, Poisoning and Procedural Complications	Off-Label Use	2586	6.72 (6.45–6.99)	6.24 (11,494.83)	6.22 (6.02)	2.64 (2.58)
		Product Dose Omission Issue	835	7.47 (6.98–8.01)	7.3 (4536.79)	7.27 (6.87)	2.86 (2.76)
		Intentional Product Misuse	239	5.37 (4.73–6.1)	5.34 (840.56)	5.32 (4.78)	2.41 (2.22)
		Contusion	150	3.03 (2.58–3.56)	3.02 (203.25)	3.02 (2.64)	1.6 (1.36)
	Musculoskeletal and Connective Tissue Disorders	Rheumatoid Arthritis	176	3.06 (2.64–3.55)	3.05 (241.99)	3.04 (2.69)	1.61 (1.39)
	Skin and Subcutaneous Tissue Disorders	Urticaria	229	2.77 (2.43–3.15)	2.76 (256.44)	2.75 (2.47)	1.46 (1.27)
canakinumab	Gastrointestinal Disorders	Abdominal Pain	253	2.35 (2.08–2.66)	2.34 (194.39)	2.34 (2.11)	1.22 (1.04)
	General Disorders and Administration Site Conditions	Pyrexia	1291	8.26 (7.81–8.73)	7.92 (7821.15)	7.89 (7.53)	2.98 (2.9)
		Malaise	694	3.37 (3.13–3.64)	3.32 (1128.56)	3.31 (3.11)	1.73 (1.62)
		Condition Aggravated	647	4.91 (4.54–5.31)	4.82 (1963.02)	4.81 (4.51)	2.27 (2.15)
		Illness	243	6.61 (5.83–7.5)	6.56 (1143.5)	6.54 (5.89)	2.71 (2.52)
	Infections and Infestations	Pneumonia	339	2.3 (2.07–2.56)	2.29 (245.99)	2.28 (2.09)	1.19 (1.03)
		COVID-19	259	3.13 (2.77–3.54)	3.11 (372.35)	3.11 (2.81)	1.64 (1.46)
		Nasopharyngitis	232	2.77 (2.43–3.15)	2.75 (259.46)	2.75 (2.47)	1.46 (1.27)
		Influenza	187	3.82 (3.31–4.41)	3.8 (385.8)	3.79 (3.36)	1.92 (1.71)
		Infection	166	2.55 (2.19–2.97)	2.54 (154.75)	2.54 (2.23)	1.34 (1.12)
	Injury, Poisoning, and Procedural Complications	Inappropriate Schedule of Product Administration	904	13.16 (12.31–14.06)	12.77 (9761.68)	12.69 (12)	3.67 (3.57)
		Incorrect Dose Administered	491	5.33 (4.87–5.82)	5.25 (1690.72)	5.24 (4.86)	2.39 (2.26)
		Product Use in Unapproved Indication	234	2.3 (2.02–2.62)	2.29 (170.84)	2.29 (2.06)	1.2 (1.01)
	Musculoskeletal and Connective Tissue Disorders	Arthralgia	502	2.66 (2.43–2.9)	2.63 (508.46)	2.62 (2.44)	1.39 (1.26)
		Joint Swelling	144	2.6 (2.21–3.07)	2.59 (141.1)	2.59 (2.26)	1.37 (1.13)
	Respiratory, Thoracic, and Mediastinal Disorders	Cough	304	2.38 (2.13–2.67)	2.37 (240.56)	2.36 (2.15)	1.24 (1.08)
		Oropharyngeal Pain	147	3.42 (2.91–4.03)	3.41 (250.45)	3.41 (2.97)	1.77 (1.53)
		Rhinorrhea	137	4.68 (3.96–5.54)	4.66 (393.85)	4.66 (4.04)	2.22 (1.97)
	Skin and Subcutaneous Tissue Disorders	Rash	454	2.33 (2.12–2.55)	2.3 (337.37)	2.3 (2.13)	1.2 (1.07)
rilonacept	General Disorders and Administration Site Conditions	Injection Site Erythema	67	21.13 (16.54–26.99)	20.29 (1230.81)	20.28 (16.53)	4.34 (3.98)
		Injection Site Pain	44	5.86 (4.34–7.9)	5.73 (172.41)	5.73 (4.46)	2.52 (2.08)
		Chest Pain	42	8.44 (6.21–11.46)	8.24 (268.11)	8.24 (6.38)	3.04 (2.6)
		Injection Site Reaction	34	19.01 (13.54–26.71)	18.64 (567.75)	18.63 (14.02)	4.22 (3.73)
		Injection Site Rash	29	38.26 (26.49–55.24)	37.59 (1032.11)	37.55 (27.61)	5.23 (4.7)
		Injection Site Pruritus	25	14.37 (9.68–21.34)	14.16 (306.07)	14.16 (10.17)	3.82 (3.25)
		Injection Site Mass	22	23.03 (15.12–35.08)	22.73 (456.92)	22.71 (15.97)	4.51 (3.9)
		Chest Discomfort	20	7.47 (4.81–11.61)	7.39 (110.68)	7.39 (5.11)	2.89 (2.25)
		Injection Site Hemorrhage	15	7.25 (4.36–12.06)	7.19 (80.08)	7.19 (4.7)	2.85 (2.12)
		Injection Site Swelling	14	7.21 (4.26–12.21)	7.16 (74.28)	7.16 (4.61)	2.84 (2.09)
		Injection Site Bruising	12	5.92 (3.35–10.45)	5.88 (48.69)	5.88 (3.66)	2.56 (1.75)
		Chills	12	3.77 (2.14–6.65)	3.75 (24.21)	3.75 (2.33)	1.91 (1.1)
		Injection Site Warmth	12	21.01 (11.9–37.07)	20.86 (226.81)	20.85 (12.96)	4.38 (3.58)
		Illness	8	3.75 (1.87–7.52)	3.74 (16.08)	3.74 (2.09)	1.9 (0.94)
		Injection Site Urticaria	8	12.98 (6.48–26)	12.92 (87.99)	12.92 (7.22)	3.69 (2.73)
	Infections and Infestations	COVID-19	19	4 (2.54–6.28)	3.96 (42.19)	3.96 (2.71)	1.99 (1.34)
	Injury, Poisoning, and Procedural Complications	Product Dose Omission Issue	55	9.49 (7.25–12.41)	9.2 (403.2)	9.19 (7.34)	3.2 (2.81)
	Nervous System Disorders	Hypoaesthesia	15	3.69 (2.22–6.13)	3.66 (29.07)	3.66 (2.39)	1.87 (1.15)
	Product Issues	Product Complaint	9	15.3 (7.94–29.46)	15.22 (119.56)	15.21 (8.79)	3.93 (3.01)

CI: confidence interval; EBGM05: lower limit of the 95% two-sided CI, for empirical Bayes geometric mean; IC025: lower limit of the 95% two-sided CI, for the information component.

**Table 3:** Contingency table for signal detection.

	Target Adverse Drug Event	Other Adverse Drug Event	Sums
IL-1 inhibitors (anakinra, canakinumab, rilonacept)	a	b	a + b
Other drugs	c	d	c + d
Sums	a + c	b + d	a + b + c + d

**Table 4:** Overview of the main algorithms used for signal detection.

Method	Equation	Criteria
ROR	ROR = $\frac{\mathrm{ad}}{\mathrm{bc}}$ ROR 95%CI = e^ln(ROR) ± 1.96^$\sqrt{\frac{1}{a}+\frac{1}{b}+\frac{1}{c}+\frac{1}{d}}$	lower limit of 95% CI > 1, *N* ≥ 3
PRR	PRR = $\frac{a(c+d)}{c(a+b)}$ PRR 95%CI = e^ln(PRR) ± 1.96^$\sqrt{\left(\frac{1}{a}-\frac{1}{a+b}+\frac{1}{c}-\frac{1}{c+d}\right)}$ χ2 = [(ad − bc)^2](a + b + c + d)/[(a + b)(c + d)(a + c)(b + d)]	lower limit of 95% CI > 1, *N* ≥ 3
BCPNN	IC = log_2_$\frac{a(a+b+c+d)}{\begin{array}{c} \left(a+b)(a+c\right) \end{array}}$ IC_025_ = e^ln(IC) − 1.96^$\sqrt{\left(\frac{1}{a}+\frac{1}{b}+\frac{1}{c}+\frac{1}{d}\right)}$	IC025 > 0
MGPS	EBGM = $\frac{a(a+b+c+d)}{\begin{array}{c} \left(a+c)(a+b\right) \end{array}}$ EBGM05 = e^ln(EBGM) ± 1.96^ $\sqrt{\frac{1}{a}+\frac{1}{b}+\frac{1}{c}+\frac{1}{d}}$	EBGM05 > 0

## Data Availability

The FDA Adverse Event Reporting System (FAERS) database and source are freely available: openFDA is freely accessible at https://api.fda.gov/drug/event.json, accessed on 1 January 2025. OpenVigil FDA can be used or downloaded at http://openvigil.sourceforge.net, accessed on 1 January 2025.

## Supplementary Materials

The following supporting information can be downloaded at: https://www.mdpi.com/article/10.3390/ph18121827/s1. Table S1: The system organ classes (SOCs) reported in IL-1 inhibitor-related AEs; Table S2: The IL-1 inhibitor-related AEs that satisfied all four criteria concurrently; Table S3: The IL-1 inhibitor-related AEs that satisfied all four criteria concurrently in different gender; Table S4: The IL-1 inhibitor-related AEs that satisfied all four criteria concurrently in different age; Table S5: The IL-1 inhibitor-related AEs that satisfied all four criteria concurrently in different reporter type; Table S6: The detailed narrative review of individual case reports for postural orthostatic tachycardia syndrome (POTS) associated with anakinra and pulmonary valve incompetence (PVI) associated with rilonacept.

## Abbreviations

AEs	Adverse Events
BCPNN	Bayesian Confidence Propagation Neural Network
CAPS	Cryopyrin-Associated Periodic Syndromes
DEMO	Demographic and Administrative Information
DRUG	Drug Information
FAERS	FDA Adverse Event Reporting System
FDA	U.S. Food and Drug Administration
IL-1	Interleukin-1
IL-1R1	IL-1 Receptor Type 1
INDI	Indications for Drug Administration
MAPK	Mitogen-Activated Protein Kinase
MedDRA	Medical Dictionary for Regulatory Activities
MGPS	Multi-item Gamma Poisson Shrinker
NF-κB	Nuclear Factor-Kappa B
OUCT	Patient Outcomes Information
PRR	Proportional Reporting Ratio
PS	Primary Suspected
PT	Preferred Term
RA	Rheumatoid Arthritis
REAC	Adverse Drug Reaction Information
ROR	Reporting Odds Ratio
RPSR	Reported Sources
SOCs	System Organ Classes
sJIA	Systemic Juvenile Idiopathic Arthritis
THER	Drug Therapy Start Dates and End Dates
TRAPS	Tumor Necrosis Factor Receptor-Associated Periodic Syndrome
