# Supplementary material for: Disproportionality Analysis of Adverse Events Associated with IL-1 Inhibitors in the FDA Adverse Event Reporting System (FAERS)
Source: Pharmaceuticals (Basel). 2025 Dec 1;18(12):1827. doi: 10.3390/ph18121827 (PMC12736109; doi:10.3390/ph18121827)
Supplement: Supplementary file 1 [file pharmaceuticals-18-01827-s001.zip › Supplementary Tables.pdf]

Supplementary Tables

Supplementary Table S1. The system organ classes (SOCs) reported in IL-1 inhibitor-related AEs

Supplementary Table S2. The IL-1 inhibitor-related AEs that satisfied all four criteria concurrently

Supplementary Table S3. The IL-1 inhibitor-related AEs that satisfied all four criteria concurrently in different gender

Supplementary Table S4. The IL-1 inhibitor-related AEs that satisfied all four criteria concurrently in different age

Supplementary Table S5. The IL-1 inhibitor-related AEs that satisfied all four criteria concurrently in different reporter type

Supplementary Table S6. The detailed narrative review of individual case reports for postural orthostatic tachycardia syndrome (POTS) associated with anakinra and pulmonary valve incompetence (PVI) associated with rilonacept.
